# Web-Based Coping Skills Training and Coach Support for Women Living With a Partner With an Alcohol Use Disorder: Randomized Controlled Trial

**DOI:** 10.2196/56119

**Published:** 2024-08-29

**Authors:** Robert G Rychtarik, Brian G Danaher, Neil B McGillicuddy, Milagra S Tyler, Christopher Barrick, Florence Leong, Derek B Kosty

**Affiliations:** 1 Department of Psychiatry and Clinical and Research Institute on Addictions University at Buffalo, The State University of New York Buffalo, NY United States; 2 Prevention Science Institute University of Oregon Eugene, OR United States; 3 Clinical and Research Institute on Addictions University at Buffalo, The State University of New York Buffalo, NY United States; 4 School of Nursing University at Buffalo, The State University of New York Buffalo, NY United States

**Keywords:** alcohol use disorder, AUD, spouses, coping skills training, eHealth, coaching, telehealth, web-based, women, randomized controlled trial, RCT, mobile phone

## Abstract

**Background:**

Individuals living with a partner with an alcohol use disorder (AUD) can experience significant psychological distress and use health care more than those without a partner with an AUD. However, the prevailing treatment system’s focus on the partner and personal barriers limit these individuals from getting help for themselves. Preliminary work on a self-directed, web-based coping skills training program, *Stop Spinning My Wheels* (SSMW), shows promise in broadening available treatments for this population. In this study, we conducted a robust evaluation of SSMW primary outcomes.

**Objective:**

The study aims to test whether women with a partner with an AUD assigned to SSMW experienced a greater reduction in negative affect (depression and anger) (1) than a usual web care (UWC) control and (2) with brief phone coach support (SSMW+coach) rather than without (SSMW only) and (3) whether baseline negative affect moderated treatment effects.

**Methods:**

Women (mean age 45.7, SD 10.8 years; Black: 17/456, 3.7%; White: 408/456, 89.5%) were randomized to SSMW only, SSMW+coach, or UWC. Depression (Beck Depression Inventory–II) and anger (State-Trait Anger Expression Inventory 2–State Anger) were assessed at baseline, 12-week posttest, and 6- and 12-month follow-ups.

**Results:**

Participants in all conditions decreased in depression from baseline to posttest and from baseline to follow-up; SSMW-only and SSMW+coach participants decreased in anger, but UWC participants did not. Compared to UWC participants, SSMW-only participants experienced greater anger reduction (*P*=.03), and SSMW+coach participants experienced a greater reduction in depression (*P*<.001) from baseline to posttest. However, from baseline to follow-up, only a greater, but not statistically significant (*P*=.052), reduction in anger occurred in SSMW+coach compared to UWC. Although the SSMW conditions did not differ from each other in negative affect outcomes (*P=*.06-.57), SSMW+coach had higher program engagement and satisfaction (all *P*<.004). Baseline negative affect did not moderate effects, although remission from baseline clinically relevant depressive symptoms (Beck Depression Inventory≥14) was higher in SSMW only (33/67, 49%; odds ratio 2.13, 95% CI 1.05-4.30; *P=*.03) and SSMW+coach (46/74, 62%; odds ratio 3.60, 95% CI 1.79-7.23; *P*<.001) than in UWC (21/67, 31%); remission rates did not differ between the SSMW conditions (*P=*.12).

**Conclusions:**

The results partially supported the hypotheses. The SSMW conditions had earlier effects than UWC, but positive change in UWC mitigated the hypothesized long-term SSMW-UWC differences. The results highlight the importance of incorporating active controls in web-based clinical trials. Although SSMW+coach showed benefits over SSMW only on engagement and satisfaction measures and in the number needed to treat (5.6 for SSMW only; 3.2 for SSMW+coach), the SSMW conditions were comparable and superior to UWC on depressive symptom remission levels. Overall, SSMW with or without a coach can reduce clinically meaningful distress and add to available treatment options for this large, underserved group.

**Trial Registration:**

ClinicalTrials.gov NCT02984241; https://www.clinicaltrials.gov/study/NCT02984241

## Introduction

### Background

Individuals living with a partner with an alcohol use disorder (AUD) are at significantly greater risk of experiencing physical or emotional harm from the partner’s drinking. Women are more likely than men to report such harm [1], and estimates suggest that 5% of adult women in the United States are married to or living with a partner with an AUD [2]. Spouses with (vs without) a partner with a substance use disorder (SUD) are at least 2 times as likely to experience depression, anxiety, and stress-related disorders [3] and have more social adjustment problems [4], subclinical disorders [5], and health care use [3,6-11] and higher health care costs [8]. While early views interpreted the psychological problems experienced by this population as indicative of personality and characterological issues, contemporary family stress models view it as indicative of normal individuals struggling to cope with the disorder in their partner or family [12,13]. Consistent with this latter view, much of the distress experienced particularly among those without their own SUD appears directly related to stress, burden, or the extent of problems brought on by the partner’s SUD [12,14,15].

In this research report, we refer to *partner* as the person with the AUD; *spouse* refers to the individual married to or living with the person with the AUD. Daley et al [16] note that the “major health, social, and safety problem [of SUD] cannot be effectively addressed without considering the impact of SUDs on families and members, including children, and including them in treatment and recovery. While addiction is promoted as a ‘family disease,’ in reality many family members are not offered the opportunity to engage in treatment for their own health.” A small but growing body of research suggests that spouses of partners with AUD can benefit from professional, therapist-delivered treatments designed to relieve their own personal distress [17,18]. However, institutional barriers (eg, systemic emphasis on the partner’s treatment) and personal barriers (eg, spouse preoccupation with only the partner’s problem, fears of partner retribution, stigmatization, and costs of clinical care) limit the overall accessibility of these face-to-face treatments. Self-help groups, mainly Al-Anon, can be helpful for this group, but Al-Anon may not be readily available, and dropout can be high [19]. Merkouris et al [17] recommended developing and evaluating a broader range of treatment modalities and intensities for spouses and caregivers, particularly self-directed, web-based treatment formats.

Only 2 studies, both pilots, of web-based treatments for spouses have been reported in the literature. Osilla et al [20] piloted a web adaptation of the Community Reinforcement and Family Training program that targeted spouses whose partners were military service members and veterans. Compared to a waitlist control condition, program participants reported significantly reduced anxiety and greater social support but no significant reduction in depressive symptoms or anger. In a feasibility pilot study, Rychtarik et al [21] conducted a randomized controlled trial (RCT), in which 89 women with a partner with an AUD were assigned to either (1) an early version of the web-based *Stop Spinning My Wheels* (SSMW) program, a coach-assisted eHealth adaptation of an empirically tested face-to-face coping skills training program focusing on spouse functioning [5]; or (2) a waitlist control. The results of an 8-week assessment of SSMW showed that women participated in their web sessions and were very satisfied with the overall program, although relatively few opted to use the optional coach support available to them. Compared to controls, SSMW participants exhibited significantly improved coping skills (effect size=1.02), fewer depressive symptoms (effect size=–0.65), and reduced situational anger (effect size=–0.70). The conditions did not differ on other secondary outcomes (ie, anxiety, anger expression, and general stress). While results are promising, we do not know whether SSMW can achieve similar results when evaluated against an active control condition. Similarly, we do not know whether coach support can improve outcomes when compared to SSMW alone.

### Objectives

In this report, we build on and address the limitations of our earlier SSMW pilot with an updated SSMW version, a credible active web control, and randomization to phone coach support. This report is limited to the study’s primary treatment outcomes (ie, depression and state anger) and to testing the study’s 4 primary a priori hypotheses. First, we hypothesized that the fully self-directed SSMW would result in immediate and sustained reductions in negative affect relative to an active web control. Second, we hypothesized that SSMW plus low-intensity phone coaching would result in immediate and sustained negative affect reductions relative to the active control. Third, we hypothesized that SSMW with low-intensity phone coaching would promote a greater negative affect reduction than SSMW alone. Fourth, we hypothesized that baseline negative affect would moderate SSMW outcomes such that those higher in negative affect would show a greater reduction with a coach than without. Those lower in negative affect were hypothesized to benefit equally well regardless of coach support. Similarly, we explored whether baseline negative affect moderated the effects of SSMW overall compared to the usual web care (UWC) control condition. Hypotheses 3 and 4 are informed by the supportive accountability model [22], which posits that low-intensity human coach support, goal setting, and performance monitoring can encourage web-based program follow-through. Thus, we hypothesized that the greater SSMW program engagement and use of program recommendations with a coach would result in a greater negative affect reduction than SSMW without a coach. Similarly, we hypothesized that the greater program engagement with a coach would promote greater benefit for those high in negative affect; those with lower negative affect would benefit from either SSMW condition.

## Methods

### Trial Design

This study was a parallel 3-group, intention-to-treat RCT that followed the CONSORT (Consolidated Standards of Reporting Trials) guidelines [23] (Multimedia Appendix 1). The trial was registered at ClinicalTrials.gov (NCT02984241) [24].

### Ethical Considerations

All study procedures were approved by the institutional review board of the University at Buffalo (STUDY00000057). Electronic and verbal consent were used in the consenting process. Participants could opt out of taking part at any time. Data presented in this paper are deidentified; only grouped summary statistics are reported. Participants were paid US $120 at week 12 for completing the interim and 12-week assessments and US $50 for each 6- and 12-month assessment.

### Participants

#### Overview

**Figure 1:**
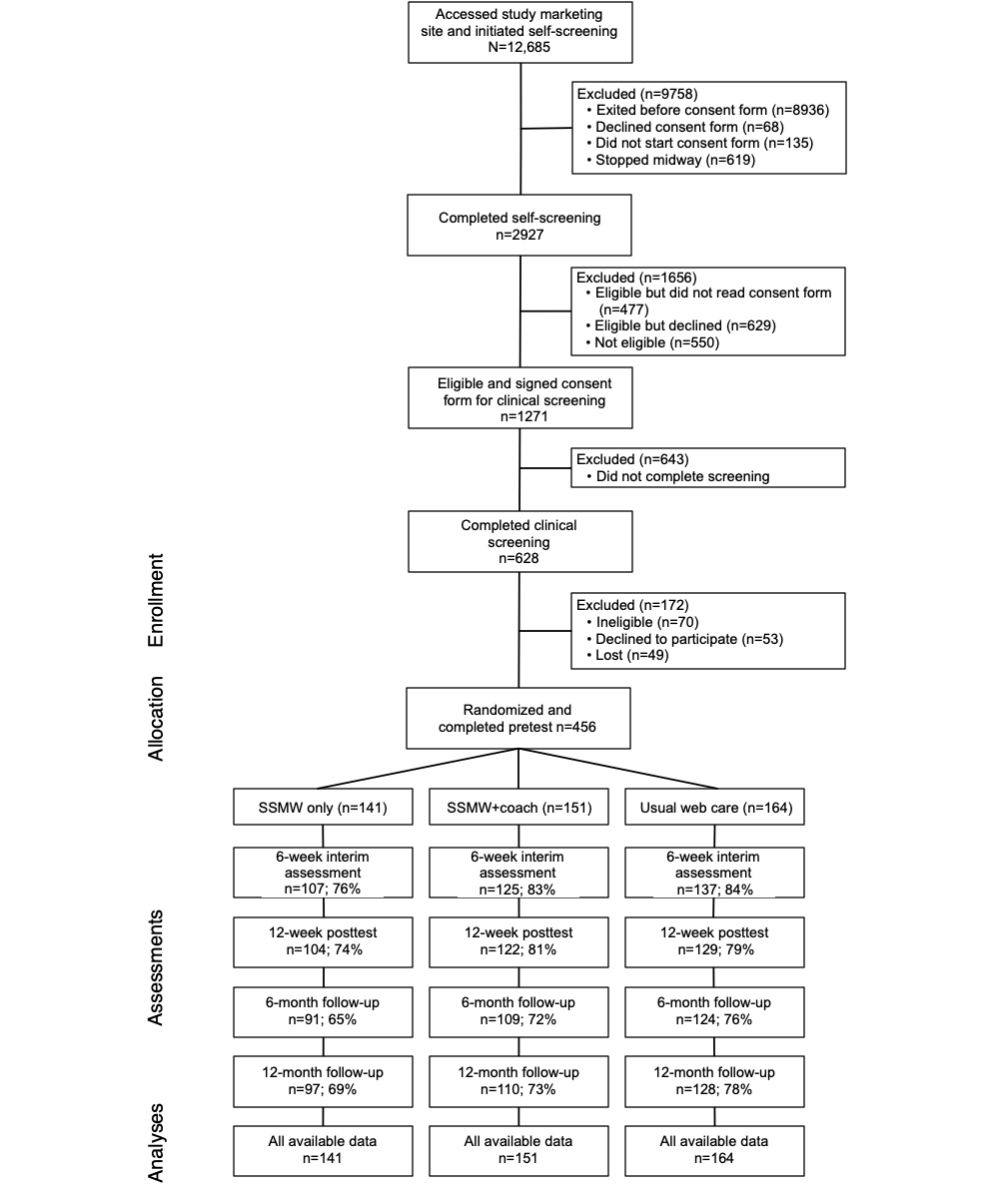
CONSORT (Consolidated Standards of Reporting Trials) participant flow diagram. SSMW: Stop Spinning My Wheels.

Study participants were 456 female spouses recruited from across New York state from October 13, 2019, to February 26, 2021. Radio, internet, and other media advertisements for the SSMW program directed women experiencing stress from their partner’s drinking to a research project web page that contained a study description and a link to a brief web self-screening consent form and questionnaire. Spouses who completed the self-screening assessment, were found to be preliminarily eligible, and consented to proceed to the next step in screening were able to access a secure web portal to schedule a call with study staff to complete their final screening (Figure 1).

Upon completion of the final screening interview, eligible and interested individuals received log-in credentials to the study’s secure main web consent form, which, when endorsed, enabled them to complete a web-administered baseline assessment and be randomized to a condition. Once randomized, participants accessed their assigned condition without charge. Throughout the screening process, individuals found ineligible or who declined to participate were provided with information on alternative treatment resources.

#### Recruitment Deviation

At 5 months into participant recruitment, at the onset of the COVID-19 pandemic, we paused prospective participant screenings for approximately 3 months to prepare for and accommodate study personnel’s work from remote locations (eg, acquiring equipment and establishing secure remote procedures). During this time, the marketing website informed visitors of the pause in recruitment and invited them to take the web-based self-screening and, if eligible, place themselves on a waiting list for later consideration. During this time, previously randomly assigned participants maintained access to their assigned study condition and, if assigned, coach phone support.

#### Eligibility

Eligibility criteria for spouses included the following: (1) age of ≥18 years; (2) residence in New York state; (3) fluency in English; (4) a woman married to or living with a male partner and, based on her report, the partner meeting criteria for past-year AUD on the Family History Assessment Module [25] and past 3-month problematic alcohol consumption, determined by a score of ≥5 on the Alcohol Use Disorders Identification Test–Consumption items [26]; (5) negative screening on the Structured Clinical Interview for the Diagnostic and Statistical Manual of Mental Disorders, Fifth Edition [27] for any unremitted SUD or serious mental disorder (ie, schizophrenia or bipolar disorder) or other conditions that would interfere with participation (eg, high suicide risk); (6) no participation in previous SSMW studies; (7) negative report of having immediate fear for her life due to intimate partner violence and no experienced injuries from partner violence requiring medical attention over the previous year; and (8) internet access and a computer or tablet and smartphone.

#### Randomization

On completion of the baseline assessments, an urn randomization procedure [28] was used to assign each participant to 1 of 3 conditions: SSMW only, SSMW+coach, or a UWC control condition. Urn randomization was used to balance condition assignment on putative prognostic baseline variables: (1) spouse education (<12 years vs ≥12 years); (2) partner SUD treatment history (last 3 months: yes vs no); (3) spouse recent Al-Anon experience (last 3 months: yes vs no); (4) spouse psychological or mental health counseling (last 3 months: yes vs no); (5) spouse depression status (<14 vs ≥14 baseline score on the Beck Depression Inventory–II [BDI-II] [29]); (6) spouse anger status (<18 vs ≥18 baseline score on the State Anger subscale of the State-Trait Anger Expression Inventory 2 [STAXI-2] [30]); and (7) age of youngest biological, adopted, or stepchild at home (none vs 1.5-5 years vs 6-18 years). BDI-II and STAXI-2 subscale cut points correspond to mild depression and clinically significant anger, respectively [29,30]. A separate urn procedure was used to randomize participants in the SSMW+coach condition to 1 of 2 available coaches.

### Procedure

#### Overview

Once randomized to a condition, participants were sent an automated welcoming email with a URL to create a password to access their assigned condition home page. All conditions shared a standard SSMW logo, name, and style (font and color palette) and were accessible via desktop, laptop, or tablet computer devices. Participants were encouraged to complete their assigned program during the *active treatment period*, defined as from randomization to 12 weeks. Study participants could subsequently access their web content during the follow-up period to more accurately reflect the design of many real-world internet treatments.

#### SSMW-Only Condition

Both SSMW conditions shared the overarching road trip theme that encouraged participants to (1) view living with a partner with an AUD as a particular challenge in one’s *road trip of life*; (2) recognize that it is not possible to be completely prepared to deal with this challenge; and (3) understand that usual ways of coping with a partner’s drinking problem often do not help and the failure of repeatedly trying to change a partner’s behavior can cause the spouse to feel exhausted and stressed out, such as *spinning their wheels*. Similarly, the stated overall goal of both SSMW conditions was to help the spouse reduce (better manage) her stress by providing more effective skills to cope with a partner who drinks too much.

The SSMW program was an enhanced 20-session version of the self-directed SSMW program in the study by Rychtarik et al [21]. The information architecture [31,32] of SSMW defined an overall step-by-step (session-by-session) path used by study participants as they interacted with the program. Participants initially followed this tunneled approach that involved completing one session at a time in order. Participants were encouraged to interact with all content on each session web page (eg, finish each video or choose the best response from several possible video interactions) before moving to a subsequent web page. Specifically, SMS text message reminders to complete activities were used throughout, and web page *next* buttons were dormant the first 2 times a user attempted to move to the next web page before she completed all activities. After 2 tries, the *next* button was enabled, and the user was able to move forward. Finally, SSMW participants could freely access the content of any sessions they had previously completed. As a result, we describe the SSMW program as having a hybrid information architecture.

Program goals centered on training participants to use strategies and *Rules of the Road* for coping more effectively with stressful problems arising from their partner’s drinking. Informed by the family stress and family interactional model [12,14,33] and using web-delivered adaptations drawn from cognitive behavioral therapy [34,35], these strategies included shifting one’s focus to their own needs, managing negative thinking, adjusting expectations for change, using functional analysis to understand self- and partner behavior, and developing more effective problem-solving and communication skills.

Each session included a video host-delivered overview complemented by narrated animations, instructional text, and multiple highly realistic video portrayals of 6 women and their partners interacting in multiple real-world scenarios of stressful problems arising from the partner’s drinking. Extracted video screenshots with their related text instructions can be found in Multimedia Appendix 2. Structured, interactive exercises and end-of-session journaling encouraged participants to review their personal problems with their partner’s drinking and apply new skills to better manage these problems. Subsequent sessions guided the participant to develop long-term personal goals and, if interested, seek additional help through professional resources and self-help groups. SSMW participants also had 24/7 secure access to an app on their smartphones [31] that provided a repository of tailored SSMW program videos, library articles, and personalized journal content synchronized to the participant’s place in the program. The SSMW program protected user privacy in part by timing out (logging the user out of the program and displaying its log-in screen) after 60 minutes of user inactivity, defined as no clicking or typing on the web page. SSMW SMS text messages were automatically sent to participants throughout the 12 weeks. Some messages were prompted by periods of participant inactivity (eg, “Haven’t seen you lately. Spun out? Just get back on the road again. Lots more useful information awaits you.”), by relevance to specific SSMW sessions (eg, “Dial down strong negative feelings by managing your thoughts—watch out for Awful, All or nothing, or Poor-me thoughts.”), and when progress milestones were achieved (eg, “Stop the Spinning! You’ve got a good start!”).

#### SSMW+Coach Support

Content of the SSMW+coach condition was essentially the same as that described for the SSMW-only condition but with the addition of 6 scheduled support calls with an assigned SSMW coach during the initial 12-week active treatment period. Coach reminder prompts were also included in the SSMW program content, and selected SMS text messages encouraged participants to reach out to their phone coach for more help and additional phone sessions if needed. Automated emails with coach call appointment confirmations also assisted in follow-through.

While the SSMW web program provided the primary coping skills training, SSMW’s phone coaching was informed by the supportive accountability model [22] and was designed to (1) support and promote spouse motivation to adhere to the program and avoid dropout and (2) offer adjunctive training as needed. Coach calls addressed motivation and dropout by dealing with practical log-in, navigation, or other problems that might discourage engagement; praising engagement; and bolstering the support and connection associated with working along with the SSMW program. Adjunctive skill training assessed the participant’s understanding of program content and its applicability to the participant’s life. As needed or requested by the participant, the coach could provide additional direct instruction, modeling, and role-play to help bolster and generalize the skills learned. Coach calls were designed to be no longer than 20 minutes. However, calls could last longer depending on each participant’s engagement level and understanding of program content and whether problems or questions were elicited during the call. To prepare for a call, the coach used a separate coach web portal to review participant progress in completing program sessions and journal entry content.

Coaches in the SSMW+coach condition were 2 master’s-level substance abuse or mental health counselors. As part of the coach fidelity and credibility protocol, coaches received intensive training in the SSMW content and phone coaching before the start of recruitment. A written phone coach manual described a framework for coaching, critical coaching skills within a supportive accountability model [22] (eg, effective use of empathic and reflective listening, eliciting commitment, use of praise, and engaging the participant collaboratively), and troubleshooting coaching issues (eg, dealing with low program adherence). In addition, to maintain consistency and minimize drift, coaches used a phone coach call checklist, digitally recorded coaching calls, and attended weekly or biweekly joint review or booster training sessions with a project supervisor to review select call recordings and discuss the status of cases. We reviewed randomly selected coach call progress notes on study completion to assess coach compliance and call recording lengths. One call was randomly chosen from each individual in a 30.1% (40/133) random sample of SSMW+coach participants who had at least one coach call; due to technical recording errors, 2 participants’ call length data were unavailable. In total, 2 raters independently scored each progress note narrative on the presence (1=yes; 0=no) of the following call compliance indicators: (1) assessment of program progress, (2) engagement or encouragement, (3) help with the application of SSMW content, and (4) a cognitive behavioral versus other counseling model focus. Interrater reliability (intraclass correlation coefficient; 2-way random model for rater consistency and mean rater measurement) was 0.77. Overall compliance with the coaching protocol appeared high. Across the 2 raters, the sampled notes averaged 3.6 (SD 0.5) out of 4 compliance indicators; the median was 4 (IQR 3.5-4.0). The mean length of calls was 20.0 (SD 9.2) minutes; the median was 19.9 (IQR 11.9-25.3) minutes.

#### UWC Condition

Women assigned to the UWC condition received access to a text-based, menu-driven site with limited still graphics. Unlike the SSMW conditions, the program presented verbatim or paraphrased content from web searches of freely accessible information on how to deal with a partner’s drinking problem. However, unlike typical search result listings, the UWC site repackaged and organized search results into appealing, meaningful subject areas and adhered to a nonlinear site architecture (ie, open to exploration). The main clickable menu topic areas included information about alcohol, AUDs, the effects of the partner’s drinking on the family (her and the children, if applicable), ways to cope, and information about treatment for him and on seeking more help for herself. In addition, the site provided URL links to obtain more information from external websites (eg, Al-Anon and the National Institute on Alcohol Abuse and Alcoholism). UWC participants were able to access their program content in a 14–web page freestanding website organized into 4 major sections (*Welcome* and *About the program*, *About alcohol problems*, *His drinking and you*, and *What can you do?*). Introductory UWC material informed the participant that, while the web contains a broad range of helpful information, it is not readily available in one spot and requires a lot of work to identify. So, to make the information more accessible, the site did the web search work for her, compiling the latest content from the internet and organizing it for her use. A screenshot of the UWC introductory page can be found in Multimedia Appendix 3.

In the first 2 weeks of enrollment, program use and encouragement SMS text message prompts in the UWC condition coincided in number and type with those in the SSMW conditions. Additional encouragement SMS text messages at 6 and 12 weeks were yoked to the estimated schedule of similar messages sent to participants in the SSMW conditions. Inactivity SMS text messages were sent on the same schedule to both SSMW conditions.

We chose this UWC condition to address several challenges of incorporating credible, active control conditions in web-based intervention research [36]. Specifically, (1) the UWC closely approximated *usual care* on the web (ie, access to free helpful content that individuals could typically find themselves using web search engines); (2) unlike a referral to available self-help groups such as Al-Anon (face-to-face or web-based), it shared the framework of web interventions in having easy access, high reach potential, and promise for high public health impact; (3) it controlled for demand characteristics and participant expectancies better than a waitlist control condition while providing a control condition available throughout the follow-up period; and (4) by providing the UWC within a self-contained website, it was possible to track participant engagement in an automated, unobtrusive manner. The UWC content was credible and useful but did not delineate step-by-step behavioral strategies.

### Follow-Up and Measures

#### Overview

Participant demographics were measured at baseline. Participant credibility and expectancy were assessed at baseline and the 6-week interim assessment; satisfaction was assessed at the 6-week interim and the 12-week posttest. Negative affect was measured at baseline, the 12-week posttest, and the 6- and 12-month follow-ups.

#### Negative Affect Measures

Guided by the key findings of the SSMW pilot work, the 21-item BDI-II [29] was the primary measure of depressive symptom severity, and the State Anger subscale of the STAXI-2 [30] was the primary measure of anger severity. The latter scale measures anger intensity in the moment or current situation. It contrasts with the STAXI-2 Trait Anger subscale, which measures one’s overall tendency to have an angry temperament. The State Anger subscale was chosen as the most appropriate of the 2 scales in this study. Specifically, the stress and coping model of spouse functioning, on which the SSMW program is built, views the anger observed in this population as a reaction to stress and problems brought on by the partner’s drinking (ie, state anger) and not the sign of an inherently angry person (trait anger). As in the pilot study, we applied a negative inverse transformation of STAXI-2 scores to reduce skew and accommodate outliers in our statistical models. Exploratory analyses used BDI-II (score of ≥14) and STAXI-2 State Anger subscale (score of ≥18) cut points to define clinically relevant levels of depression and state anger. These points correspond to mild depression and clinically significant anger cut points as defined by Beck et al [29] and Spielberger [30], respectively.

#### Participant Engagement and Coach Calls

For all conditions over the entire duration of the study, the database system used by program websites collected a set of automated primary program engagement metrics continuously and unobtrusively, including the number of visits to the website, the duration of visits made to program sessions, and the date in which sessions were completed. The coach recorded the number of sessions and content covered in scheduled coach calls. The posttest mean of the 12-item Working Alliance Inventory–Short Form (WAI-SF) [37] completed by SSMW+coach participants measured the quality and strength of collaborative engagement with the coach. WAI-SF items are rated on a 7-point scale ranging from 1 (*never*) to 7 (*always*) regarding participant and coach agreement on goals (Goals subscale) and tasks (Tasks subscale) and the perceived participant-coach bond (Bond subscale). As all subscales were highly correlated with the total mean item score (0.91-0.96), we used the latter to measure participant-coach engagement.

#### Credibility, Expectancy, and Satisfaction

We used the Credibility/Expectancy Questionnaire (CEQ) [38] at baseline before randomization and at the 6-week interim assessment to measure participant self-reported ratings of the extent to which they found their assigned program’s content credible and consistent with their preprogram expectations. Credibility scores were calculated as the mean of the first 3 items of the CEQ. Expectancy was based on the item from the CEQ that asked participants to rate the following: *By the end of the program, how much improvement in your stress do you think will occur*? Overall satisfaction with the program was measured using the Client Satisfaction Questionnaire–8 [39] at the interim assessment and the 12-week posttest.

#### COVID-19 Impact

We developed and administered a COVID-19 impact assessment at baseline using 30 items adapted from Behar-Zusman et al [40]. Items focused on the pandemic’s effect on the household (eg, *We self-quarantined due to travel or possible exposure*) and on family relationships (eg, *You spent a lot more time taking care of or trying to keep other family members occupied*). The overall score was the sum of the items, each scored as 0=no and 1=yes. The internal consistency of this measure was 0.72. This scale was used in unplanned, exploratory analyses to assess for baseline differences between conditions on pandemic impact.

### Statistical Analysis

#### Overview

We assessed condition differences in outcome gains from pretest to posttest using a mixed-model (multilevel) time × condition analysis [41] represented by the following equation:

*Y_tj_* = (γ_00_ + γ_01_*C_j_* + γ_10_*T_tj_* + γ_11_*T_tj_C_j_*) + (*r*_0_*_j_* + *r*_1_*_j_T_tj_* + *e_tj_*)

*Y_tj_* represents the outcome for assessment occasion *t* on participant *j*. The model included 3 predictors: time, denoted by *T_tj_* (coded 0 at baseline and 1 at posttest); condition, denoted by *C_j_* (eg, coded 0 for control and 1 for treatment); and their interaction. Our planned tests of condition differences included comparisons between each SSMW condition (SSMW+coach and SSMW only) and the UWC condition as well as comparisons between the SSMW+coach and the SSMW-only conditions. The model produces estimates of the baseline intercept for the comparison condition, γ_00_; the difference between conditions at baseline, γ_01_; pretest-posttest change for the comparison condition, γ_10_; and the difference in the change in outcomes between conditions, γ_11_ (treatment efficacy). The random effects account for participant-level variability in the intercept, *r*_0_*_j_*; improvements in outcome, *r_1j_T_tj_*; and the residual, *e_tj_*, in a time × condition model with 2 time points (*e_tj_*=0) due to constraints imposed by only 2 assessments.

We extended the model to include >2 time points to test for condition effects through the 12-month follow-up assessment. This extended model assumed an unstructured covariance matrix and included outcome data at baseline (time coded as 0), 6-month follow-up (time coded as 1), and 12-month follow-up (time coded as 2). We subsequently graphed descriptive statistics for outcomes by condition and assessment time to facilitate interpretation of results.

We explored the possibility that negative affect moderated treatment effects by extending the model to include baseline scores of the BDI-II and negative inverse transformations of raw baseline scores on the STAXI-2 as well as their interactions with time, condition, and the time × condition term. Pearson *r* correlation coefficients were calculated to describe the associations between the SSMW engagement metrics and outcome improvements from baseline to posttest.

#### Model Estimation

Time × condition models were estimated using the SAS (version 9.4; SAS Institute) [42] PROC MIXED procedure using full-information maximum likelihood (ML) methods. ML estimation uses all available data, reducing potential bias—even in the face of substantial attrition—provided data are missing at random [43]. Compared to complete-case analyses, ML relies on relatively benign assumptions and does not introduce bias [44,45].

#### Interpretation of Results

To supplement *P* values in our interpretation of impact results, we reported Hedges *g* effect sizes, their 95% CIs, and model probabilities based on the Akaike information criterion [46] as recommended by the American Statistical Association [47]. Model probabilities, *w*, indicate the strength of evidence for one model when compared with others given the data at hand. Burnham et al [46] described *w* as the probability of selecting the same model with a “replicate data set from the same system” and “allow statements such as the probability of [H_A_] is 0.78.” Model probabilities better characterize the chance of a replicated result than *P* values. In this study, we compared models for 2 hypotheses: a model with the effect of study condition (H_A_: alternate hypothesis) and one without (H_0_: null hypothesis). We reported the model probability for the model with the condition effect (H_A_), and with only 2 models, the model probability for H_0_ is 1 – *w*.

### Sample Size and Power

This study was designed to detect small to moderate differences (minimum detectable effect size of 0.38 SDs) between study conditions. Power analyses assumed 450 participants (exceeded by the actual sample of 456) randomly assigned to 1 of the 3 conditions, a type-II error rate of 20% (power=0.80), a type-I error rate of 0.05 for 2-tailed tests of condition, a moderate relationship between baseline and posttest outcome measures (*r*=0.50), and 5% attrition (loss to follow-up) at posttest and 20% attrition at the 12-month follow-up.

## Results

### Overview

**Figure 2:**
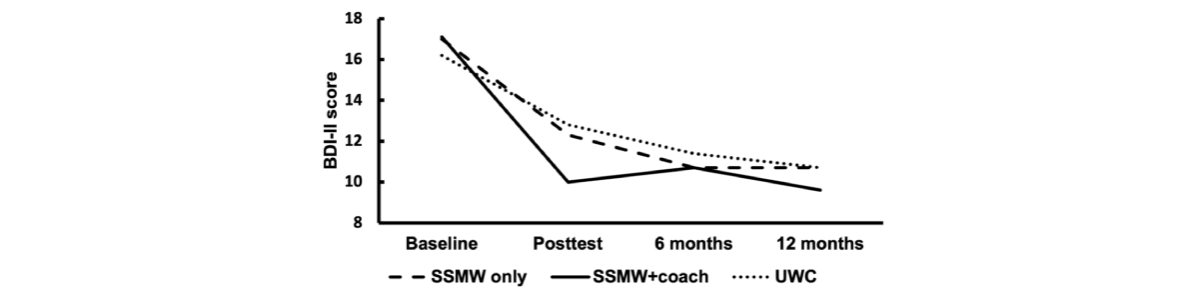
Observed mean depression scores on the Beck Depression Inventory–II (BDI-II) throughout the assessment period. SSMW: Stop Spinning My Wheels; UWC: usual web care.

**Figure 3:**
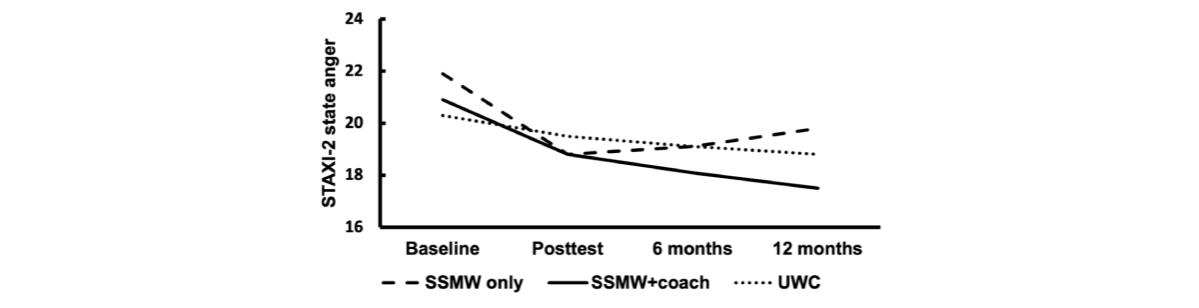
Observed mean, untransformed state anger scores on the State-Trait Anger Expression Inventory 2 (STAXI-2) throughout the assessment period. SSMW: Stop Spinning My Wheels; UWC: usual web care.

Table 1 depicts participant demographics, partner AUD characteristics, and COVID-19 impact scale scores by study condition. We found no statistically significant condition differences in baseline participant characteristics (*P*≥.11 in all cases) or outcome measures (*P*≥.08 in all cases). Figures 2 and 3 illustrate observed depressive symptom and anger outcomes, respectively, throughout the study period. Tabled descriptive statistics for primary negative affect outcomes by assessment time and study condition as depicted in Figures 2 and 3 can be found in Multimedia Appendix 4.

**Table 1:** Baseline participant demographic and partner alcohol use disorder characteristics and COVID-19 impact indexes by study condition^a^.

			SSMW^b^ only (n*=*141)		SSMW+coach (n*=*151)		UWC^c^ (n*=*164)			
**Participant characteristics**										
	Age (y), mean (SD)			45.5 (10.2)		45.9 (10.8)		45.8 (11.4)		
	Years of school completed, mean (SD)			16.3 (2.3)		16.0 (2.3)		16.0 (2.5)		
	**Race, n (%)**									
		American Indian		0 (0)		1 (0.7)		1 (0.6)		
		Asian		2 (1.4)		5 (3.3)		2 (1.2)		
		Black or African American		3 (2.1)		7 (4.6)		7 (4.3)		
		Hispanic or Latino		5 (3.5)		14 (9.3)		14 (8.5)		
		White		130 (92.2)		128 (84.8)		150 (91.5)		
		Mixed or other		6 (4.3)		10 (6.6)		4 (2.4)		
	Currently employed, n (%)			123 (87.2)		124 (82.1)		137 (83.5)		
	**Primary source of income, n (%)**									
		Own wages		39 (27.7)		43 (28.5)		39 (23.8)		
		Partner wages		12 (8.5)		25 (16.6)		25 (15.2)		
		Own and partner wages		87 (61.7)		79 (52.3)		98 (59.8)		
		Other (eg, unemployment insurance)		3 (2.1)		4 (2.6)		2 (1.2)		
	**Relationship status, n (%)**									
		Married		110 (78)		113 (74.8)		125 (76.2)		
		Cohabiting but not married		31 (22)		38 (25.2)		39 (23.8)		
	**Child age, n (%)**									
		No children		77 (54.6)		95 (62.9)		99 (60.4)		
		<18 months		20 (14.2)		19 (12.6)		18 (11)		
		1.5-5 years		38 (27)		33 (21.9)		35 (21.3)		
		6-18 years		6 (4.3)		4 (2.6)		12 (7.3)		
	Al-Anon attendance (last 3 months), n (%)			8 (5.7)		15 (9.9)		14 (8.5)		
	Mental health counseling (last 3 months), n (%)			29 (20.6)		35 (23.2)		37 (22.6)		
	COVID-19 impact score, mean (SD)			7.7 (4.0)		7.8 (3.6)		7.7 (3.5)		
**Participant-reported partner drinking characteristics**										
	AUDIT-C^d^ score at baseline, mean (SD)			9.7 (1.9)		9.3 (1.8)		9.7 (1.8)		
	FHAM^e^ score at baseline, mean (SD)			9.3 (2.7)		8.9 (2.8)		9.0 (2.8)		
	Length of drinking problem (months), mean (SD)			210.3 (136.2)		212.7 (136.2)		209.9 (144.0)		
	SUD^f^ treatment involvement (last 3 months), n (%)			9 (6.4)		8 (5.3)		10 (6.1)		
	No current legal problems, n (%)			133 (94.3)		144 (95.4)		156 (95.1)		

^a^The sample sizes for each variable may not add up to the total sample size.

^b^SSMW: Stop Spinning My Wheels.

^c^UWC: usual web care.

^d^AUDIT-C: Alcohol Use Disorders Identification Test–Consumption items.

^e^FHAM: Family History Assessment Module.

^f^SUD: substance use disorder.

### Credibility and Expectancy

Program credibility used the CEQ scale rated from 1=*Not at all useful* to 9=*Very useful*, and expectations for improvement in outcomes used a CEQ item rated on a scale from 0% to 100%. Mean credibility and expectancy ratings at baseline were similar for SSMW only (mean 6.3, SD 1.6 and mean 52.6, SD 19.9, respectively), SSMW+coach (mean 6.2, SD 1.6 and mean 52.0, SD 19.6, respectively), and UWC (mean 6.4, SD 1.6 and mean 55.5, SD 22.1, respectively). Credibility and expectancy ratings at the 6-week interim assessment stayed stable or slightly increased for the SSMW-only (mean 6.6, SD 1.9 and mean 51.4, SD 20.1, respectively) and SSMW+coach (mean 7.3, SD 1.5 and mean 61.4, SD 21.9, respectively) conditions but were lower for the UWC condition (mean 4.7, SD 2.1 and mean 38.0, SD 26.9, respectively).

### Loss to Follow-Up

Rates of failure to complete scheduled study assessments (loss to follow-up) for the SSMW-only, SSMW+coach, and UWC conditions were 26.2% (37/141), 19.2% (29/151), and 21.3% (35/164), respectively, at posttest; 35.5% (50/141), 27.8% (42/151), and 24.4% (40/164), respectively, at the 6-month follow-up; and 31.2% (44/141), 27.2% (41/151), and 22% (36/164), respectively, at the 12-month follow-up (see Figure 1 for participant flow and follow-up rates). Study participants with children (vs no children) were less likely to complete their assessments at the posttest (129/185, 69.7% vs 226/271, 83.4% completion; N=456, *χ*^2^_1_=12.8, *P*<.001), 6-month follow-up (118/185, 63.8% vs 206/271, 76%; N=456, *χ*^2^_1_=8.0, *P=*.005), and 12-month follow-up (120/185, 64.9% vs 215/271, 79.3%; N=456, *χ*^2^_1_=11.8, *P*=.001). Attrition rates did not significantly differ as a function of condition (*P*=.10-.33 in all cases), other demographic characteristics (*P*=.07-.98 in all cases), or baseline outcome measures (*P*=.16-.38 in all cases). Interactions between attrition and study condition did not predict baseline outcome scores (*P*=.37-.82 in all cases). These results provide no evidence of bias due to attrition in our tests of condition effects.

### Results of Hypothesis-Driven Research Questions

#### Research Question 1: What Was the Impact of SSMW Only Versus UWC?

The first column of Table 2 summarizes time × condition model results comparing the SSMW-only and UWC conditions on change in negative affect outcomes from baseline to posttest. SSMW-only participants achieved greater decreases than UWC participants on the STAXI-2 (*g=*–0.34, 95% CI –0.64 to –0.03; t_303_=–2.19; *P=*.03; *w=*0.79). These results suggested that the hypothesis of a difference between conditions measured using the time × condition effect fit the data. That is, the model for STAXI-2 scores that included the time × condition interaction had a considerably higher probability (*w=*0.79) than the model without the condition difference (*w=*0.21). To illustrate the fixed effects, the model estimated a baseline intercept for the UWC condition of –0.054, a baseline-to-posttest change for the UWC condition of –0.001, a difference between conditions at baseline of 0.003, and a difference between conditions in terms of the change from baseline to posttest assessment of –0.004. We found no evidence of a condition difference between SSMW only and UWC in change in BDI-II scores from baseline to posttest (*g*=–0.14, 95% CI –0.35 to 0.06; t_303_=–1.41; *P=*.16; *w*=0.49). From baseline to the 12-month follow-up, we found no evidence of differences between the SSMW-only and UWC conditions on change in BDI-II (*g*=–0.09, 95% CI –0.27 to 0.09; t_303_=–0.95; *P=*.34; *w*=0.36) or STAXI-2 (*g*=–0.06, 95% CI –0.30 to 0.15; t_303_=–0.62; *P=*.54; *w*=0.30) scores.

**Table 2:** Time × condition model results comparing conditions on negative affect improvements from baseline to posttest.

Effect or statistic		Condition contrast and negative affect outcome							
		SSMW^a^ only vs usual web care			SSMW+coach vs usual web care			SSMW+coach vs SSMW only	
		Depression (BDI-II^b^)	Anger (STAXI-2^c^)	Depression (BDI-II)		Anger (STAXI-2)	Depression (BDI-II)		Anger (STAXI-2)
Model probability (*w*)^d^		0.49	0.79	0.99		0.52	0.67		0.33
**Fixed effects, parameter estimate (SE)**									
	Intercept	16.2^e^ (0.8)	–0.054^e^ (0.001)	16.2^e^ (0.8)		–0.054^e^ (0.001)	17.0^e^ (0.8)		–0.051^e^ (0.001)
	Time	–3.4^e^ (0.7)	–0.001 (0.001)	–3.4^e^ (0.7)		–0.001 (0.001)	–4.9^e^ (0.8)		–0.006^e^ (0.001)
	Condition	0.8 (1.2)	0.003^f^ (0.002)	0.9 (1.1)		0.002 (0.001)	0.1 (1.1)		–0.001 (0.002)
	Time × condition	–1.5 (1.0)	–0.004^f^ (0.002)	–3.4^e^ (1.0)		–0.003 (0.002)	–1.9 (1.0)		0.001 (0.002)
**Variances, parameter estimate (SE)**									
	Participant-level intercept	69.3^e^ (7.3)	0.000^e^ (0.000)	63.4^e^ (6.7)		0.000^e^ (0.000)	55.3^e^ (6.4)		0.000^e^ (0.000)
	Participant-level gain	32.2^e^ (3.0)	0.000^e^ (0.000)	31.9^e^ (2.8)		0.000^e^ (0.000)	31.7^e^ (3.0)		0.000^e^ (0.000)
**Time × condition effect size and statistical significance**									
	Hedges *g* effect size (95% CI)	–0.14 (–0.35 to 0.06)	–0.34 (–0.64 to 0.03)	–0.36 (–0.57 to 0.15)		–0.22 (–0.52 to 0.07)	–0.22 (–0.45 to 0.01)		0.13 (–0.19 to 0.44)
	*P* value	.16	.03	.001		.14	.06		.44

^a^SSMW: Stop Spinning My Wheels.

^b^BDI-II: Beck Depression Inventory–II.

^c^STAXI-2: State-Trait Anger Expression Inventory 2. The negative inverses of raw STAXI-2 scores were analyzed.

^d^The model probabilities (*w*) compares the strength of evidence for a model with the time × condition effect to one without. Condition was coded such that the second group listed for each contrast served as the reference category. Time was coded as 0 for baseline and 1 for posttest. Tests of fixed effects included 303, 313, and 289 df for each contrast.

^e^*P*<.001.

^f^*P*<.05.

#### Research Question 2: What Was the Impact of the SSMW+Coach Condition Versus UWC?

The second column of Table 2 summarizes time × condition model results comparing the SSMW+coach and UWC conditions on change in negative affect outcomes from baseline to posttest. SSMW+coach participants achieved greater decreases than UWC participants in BDI-II scores (*g=*–0.36, 95% CI –0.57 to –0.15; t_313_=–3.40; *P*=.001; *w=*0.99). We found no difference between the SSMW+coach and UWC conditions in change in STAXI-2 scores from baseline to posttest (*g*=–0.22, 95% CI –0.52 to 0.07; t_313_=–1.49; *P=*.14; *w*=0.52).

From baseline to the 12-month follow-up, SSMW+coach participants achieved greater decreases than UWC participants on STAXI-2 scores (*g=*–0.24, 95% CI –0.45 to 0.00; t_313_=–1.95; *P=*.052; *w=*0.70). Though not statistically significant, the model probability with the time × condition effect (*w=*0.70) was considerably greater than the model probability without the time × condition effect (*w=*0.30), hence providing support for the hypothesized effect, albeit a small one. We found no difference between the SSMW+coach and UWC conditions on change in BDI-II scores from baseline to the 12-month follow-up (*g*=–0.15, 95% CI –0.33 to 0.03; t_313_=–1.58; *P=*.11; *w*=0.56).

#### Research Question 3: Did Adding a Coach to SSMW Reduce Negative Affect More Than SSMW Alone?

The third column of Table 2 summarizes time × condition model results comparing the SSMW+coach and SSMW-only conditions on outcome improvements from baseline to posttest. The effect of SSMW+coach versus SSMW only on BDI-II scores during this period was small and was not statistically significant at conventional levels (*g*=–0.22, 95% CI –0.45 to 0.01; t_289_=–1.86; *P=*.063; *w*=0.67). However, the model probability with the time × condition effect (*w*=0.67) was considerably greater than the model probability without the time × condition effect (*w*=0.33), hence providing support for the hypothesized effect, albeit a small one. We found no difference between the SSMW+coach and SSMW-only conditions on baseline to posttest STAXI-2 score changes (*g*=0.13, 95% CI –0.19 to 0.44; t_289_=0.76; *P=*.45; *w*=0.33). When we examined changes from baseline to the 12-month follow-up, we also found no differences between the SSMW+coach and SSMW-only conditions for BDI-II (*g*=–0.06, 95% CI –0.27 to 0.15; t_290_=–0.57; *P=*.57; *w*=0.30) or STAXI-2 (*g*=–0.15, 95% CI –0.39 to 0.09; t_290_=–1.22; *P=*.23; *w*=0.43) scores.

#### Research Question 4: Did Baseline Negative Affect Moderate Effects of Condition?

To examine whether baseline negative affect scores moderated condition effects on negative affect outcomes from baseline to posttest, the baseline score and its interaction with time, condition, and the time × condition term was added to the models used to address the first 3 research questions. In the comparison between SSMW only and UWC, we found no moderation effect for depression severity (BDI-II scores; 3-way interaction estimate=–0.05, 95% CI –0.25 to 0.16; t_301_=–0.44; *P=*.66; *w*=0.28) or anger (STAXI-2 scores; estimate=0.03, 95% CI –0.16 to 0.21; t_301_=0.27; *P=*.78; *w*=0.27). We also found no moderation effect in the comparison between SSMW+coach and UWC on depression severity (BDI-II scores; estimate=–0.13, 95% CI –0.33 to 0.08; t_311_=–1.23; *P=*.22; *w*=0.43) or anger (STAXI-2 scores; estimate=–0.04, 95% CI –0.23 to 0.15; t_311_=–0.43; *P=*.67; *w*=0.28). Finally, we found no evidence of moderation of the SSMW+coach effects relative to SSMW only on baseline depression (BDI-II scores; estimate=–0.08, 95% CI –0.30 to 0.13; t_288_=–0.76; *P=*.45; *w*=0.32) or anger (STAXI-2 scores; estimate=–0.10, 95% CI –0.28 to 0.07; t_288_=–1.16; *P=*.25; *w*=0.41).

### Unplanned Exploratory Analyses of Clinically Relevant Change

#### Did Remission Rates Differ by Study Condition Among Women With Clinically Relevant Negative Affect at Baseline?

Because condition differences were most robust at the posttest, we explored condition differences in rates of remission at posttest from clinically relevant levels of depressive symptoms based on a BDI-II cutoff of 14. The analysis included 208 participants with a baseline BDI-II score of ≥14 and complete posttest data (n=67, 32.2% for SSMW only; n=74, 35.6% for SSMW+coach; and n=67, 32.2% for UWC). We examined condition differences in rates of remission using contingency table analyses with chi-square test statistics and reported odds ratios (ORs) as a measure of effect size. Rates of remission based on posttest BDI-II scores were 49% (33/67), 62% (46/74), and 31% (21/67) for the SSMW-only, SSMW+coach, and UWC conditions, respectively. Participants in the SSMW-only condition were more likely than UWC participants to have remitted (33/67, 49% vs 21/67, 31%; OR 2.13, 95% CI 1.05-4.30; *P=*.03). Similarly, participants in the SSMW+coach condition were more likely than UWC participants to have remitted (46/74, 62% vs 21/67, 31%; OR 3.60, 95% CI 1.79-7.23; *P*<.001). SSMW-only and SSMW+coach remission rates did not differ (*P=*.12). From a *number needed to treat* perspective [48], the SSMW-only condition required 5.6 participants to achieve 1 remission, whereas SSMW+coach needed only 3.2 participants.

We similarly explored condition differences in rates of remission among 175 participants with elevated anger at baseline (STAXI-2 score of ≥18) and complete posttest data (n=53, 30.3% for SSMW only; n=62, 35.4% for SSMW+coach; and n=60, 34.3% for UWC). Rates of remission based on posttest STAXI-2 scores were 51% (27/53), 55% (34/62), and 40% (24/60) for the SSMW-only, SSMW+coach, and UWC conditions, respectively. No group contrasts were statistically significant (*P*≥.10 in all cases).

#### Was There Differential Onset of Clinically Relevant Negative Affect Levels Among Those Without Clinically Relevant Negative Affect at Baseline?

This analysis for depression included 147 participants with a baseline BDI-II score of <14 and complete posttest data (n=37, 25.2% for SSMW only; n=48, 32.7% for SSMW+coach; and n=62, 42.2% for UWC). Rates of onset based on posttest BDI-II scores were 14% (5/37), 6% (3/48), and 10% (6/62) for the SSMW-only, SSMW+coach, and UWC conditions, respectively. No group contrasts were statistically significant (*P*≥.26 in all cases).

We also explored condition differences in rates of onset of elevated anger. The analysis included 180 participants with a baseline STAXI-2 score of <18 and complete posttest data (n=51, 28.3% for SSMW only; n=60, 33.3% for SSMW+coach; and n=69, 38.3% for UWC). Rates of onset using posttest STAXI-2 scores were 24% (12/51), 28% (17/60), and 28% (19/69) for the SSMW-only, SSMW+coach, and UWC conditions, respectively. No contrasts were statistically significant (*P*≥.57 in all cases).

### Ancillary Engagement and Satisfaction Analyses

#### SSMW Engagement

Table 3 summarizes program engagement metrics by study condition. We examined condition differences using independent-sample, 2-tailed *t* tests and associations between engagement and outcomes using Pearson correlation coefficients. Participants in the SSMW+coach condition, compared to the SSMW-only condition, visited their program more often (t_290_=4.57; *P*<.001) and for a longer duration (t_290_=5.49; *P*<.001) and completed more program sessions (t_290_=5.79; *P*<.001).

**Table 3:** Program engagement and satisfaction by study condition.

Program engagement metric	SSMW^a^ only, mean (SD; median)	SSMW+coach, mean (SD; median)	UWC^b^, mean (SD; median)
Number of visits	8.8 (7.7; 7.0)	13.4 (9.1; 12.0)	3.6 (2.6; 3.0)
Hours on the program	5.3 (5.5; 4.3)	9.7 (7.7; 8.3)	1.2 (1.8; 0.7)
Sessions completed out of 20	9.3 (7.6; 9.0)	14.1 (6.5; 16.0)	—^c^
Number of coach calls	—	3.8 (2.1; 4.0)	—
Working Alliance Inventory total score^d^	—	5.4 (1.4; 5.9)	—

^a^SSMW: Stop Spinning My Wheels.

^b^UWC: usual web care.

^c^Not applicable.

^d^Working Alliance Inventory items were rated on a 7-point scale ranging from 1 (*never*) to 7 (*always*).

The WAI-SF score in the SSMW+coach condition was positively correlated with the total number of visits to the SSMW program (*r*=0.31; *P*=.001), hours on the program across visits (*r*=0.27; *P*=.003), number of sessions completed (*r*=0.41; *P*<.001), and number of coach calls (*r*=0.55; *P*<.001). Higher WAI-SF scores also were associated with greater decreases in BDI-II scores from baseline to posttest (*r*=–0.18; *P*=.045). Notably, across both SSMW conditions, the higher number of SSMW sessions completed was associated with greater decreases in BDI-II scores from baseline to posttest (*r*=–0.20; *P*=.002), whereas other SSMW engagement metrics were not correlated with outcome gains (*P*≥.13 in all cases).

#### UWC Engagement

Participants in the UWC condition averaged 3.6 (SD 2.6) visits to their program and 1.2 (SD 1.8) hours on the program across visits. These engagement metrics were not correlated with outcome gains from baseline to posttest (*P*≥.67 in all cases).

#### Program Satisfaction

The Client Satisfaction Questionnaire–8 used a 4-point rating scale coded as 1=*Not at all satisfied* to 4=*Very satisfied*. SSMW-only and SSMW+coach participants reported being satisfied with program features at the 6-week interim assessment (mean 3.1, SD 0.6 vs mean 3.3, SD 0.5) and the 12-week posttest (mean 3.0, SD 0.8 vs mean 3.3, SD 0.6). SSMW+coach participants, compared to SSMW-only participants, reported greater satisfaction at the 6-week interim assessment (t_229_=3.99; *P*<.001) and the 12-week posttest (t_237_=2.93; *P=*.004). UWC participants, compared to SSMW participants, were less satisfied at the 6-week interim assessment (mean 2.5, SD 0.7 vs mean 3.2, SD 0.6; t_366_=11.58; *P*<.001) and the 12-week posttest (mean 2.4, SD 0.8 vs mean 3.2, SD 0.7; t_375_=9.83; *P*<.001).

## Discussion

### Support for Original Hypotheses

Primary outcome results partially supported the hypotheses regarding the presumed benefit of the SSMW-only condition over the UWC active control. Contrary to our hypotheses, the SSMW-only and UWC conditions showed comparable reductions from baseline depressive symptoms at posttest and follow-up (Figure 2) and did not differ. However, participants with a clinically relevant baseline level of depressive symptoms were significantly more likely than UWC participants to have remitted to a nonclinical level by the posttest. As predicted, SSMW-only participants experienced a greater baseline-to-posttest decrease in anger compared to UWC participants, whose state anger decreased little from the baseline level at either the posttest or follow-up. However, a rebound in SSMW-only anger appeared to erase this advantage when outcome trajectories were compared up to the 12-month follow-up (Figure 3). Remission rates from a clinically relevant level of anger also did not differ between conditions.

The results were more consistent with hypothesized differences between SSMW+coach and UWC. As predicted, at the posttest, SSMW+coach participants displayed a larger decrease in depressive symptoms and a higher remission rate than UWC participants (Figure 2). In addition, depressive symptom reductions at posttest in SSMW+coach participants appeared to be sustained during follow-up. However, concurrent UWC changes in depressive symptoms appeared to erase differences from the changes in the SSMW+coach condition when outcome trajectories were compared up to the 12-month follow-up (Figure 2). Contrary to predictions, SSMW+coach anger reductions did not differ significantly from those in the UWC condition at posttest, or posttest anger remission. However, SSMW+coach anger decreased further up to the 12-month follow-up, and their overall reduction in anger from baseline was greater to a small degree than that of the UWC condition (Figure 3). Overall, to varying degrees, the SSMW conditions appeared to facilitate earlier improvement in negative affect outcomes than the UWC, and those initial improvements, particularly for depressive symptoms, endured until follow-up. However, the reduction in UWC depression was particularly notable and may have dampened the emergence of predicted differences from the SSMW-only condition at posttest and from the SSMW+coach condition at follow-up. Unfortunately, this study was not designed to measure whether the change in UWC resulted from regression to the mean, UWC content, participant uptake of other treatment programs, or other factors.

Contrary to our hypothesis, adding a phone coach to SSMW generated only a small additional benefit on posttest depression, but this difference did not reach conventional levels of statistical significance. The video-rich, engaging design of the SSMW program alone may have mitigated the benefits that coach involvement might have provided. Nevertheless, ancillary engagement and satisfaction analyses and exploratory comparisons of the individual SSMW conditions relative to UWC provided additional support for the benefits of a coach. Specifically, compared to SSMW only, SSMW+coach participants displayed higher levels of engagement and reported greater program satisfaction. Furthermore, when compared with UWC, SSMW+coach participants achieved a greater decrease in depressive symptoms at posttest, whereas those in SSMW only did not. Moreover, while SSMW-only participants reported a greater reduction than UWC participants in anger at posttest, only SSMW+coach participants reported greater anger reduction at follow-up. More importantly, SSMW+coach achieved the highest rate of remission from clinically relevant depressive symptoms (46/74, 62%) compared to either SSMW only (33/67, 49%) or UWC (21/67, 31%). Notably, although SSMW-only participants were >2 times as likely as UWC participants to remit to below depression threshold levels, SSMW+coach participants were >3.5 times as likely. Finally, the SSMW-only condition required 5.6 participants to achieve 1 remission, whereas SSMW+coach needed only 3.2 participants. These findings suggest that adding a coach can (1) significantly improve SSMW engagement and satisfaction, (2) result in clinically meaningful change compared to usual web use, and (3) possibly confer a small advantage over SSMW alone. The quality and strength of the participant-coach relationship may play an important role in these findings, as higher WAI-SF scores were associated with higher program engagement and a greater reduction in depressive symptoms.

We did not find support for the coach-driven hypothesis that baseline negative affect would moderate change in negative affect between the 2 SSMW conditions (ie, women with higher negative affect would benefit more from having a coach than from not having one). Although results suggest that individuals experiencing a wide range of symptom levels may benefit from SSMW conditions, we caution that our sample averaged in the mild range of depressive symptoms, and their outcomes might not generalize to individuals with severe depression.

### Comparison to Prior Work

The results support and build on our web-based SSMW pilot study and our prior work on face-to-face coping skill training for spouses that used waitlist control conditions [5,21]. The active UWC condition in this study is a significant methodological advancement over our prior work that may present a more stringent comparison based on which to gauge SSMW effects. In this respect, it is noteworthy that the size of effects in this study was approximately half that of those in the waitlist-control pilot. These findings and observed improvement in UWC during follow-up highlight the importance of active controls and longitudinal follow-ups in web-based RCTs. This study also extends our previous work by examining SSMW effects with and without brief coach support. Finally, the large sample size relative to earlier work on coping skill training with this population allowed for more powerful tests of SSMW, coaching effects, moderating effects, and exploratory analyses of clinically relevant outcomes.

### Limitations

Several study limitations may have influenced internal and external validity. First, while coaches complied with the coaching protocol and collaborative engagement was relatively high, we did not gauge overall coach skillfulness. Second, SSMW+coach participants were scheduled for 6 brief coach calls, but the optimum level of coaching required and the participants who benefit most from it are unknown. Third, although our pilot found support for anger as a primary outcome, anger measurement typically has not been included in studies of this population, and anger findings were the weakest in this study. However, feelings of anger are a common experience in this population. Future research is needed to better understand and assess anger in this group and evaluate measures of angry thoughts and feelings that are more closely associated with the stressors experienced. Fourth, our study participants were predominantly White and highly educated. More diverse samples are needed. Fifth, most of the recruitment, treatment, and follow-up occurred amid the COVID-19 pandemic. Although we found no baseline condition differences in COVID-19 impact, some participants who might otherwise not have taken part did so because they had the time to do it. Others began reevaluating their life and sought help. However, as businesses and schools reopened, follow-ups became more challenging as participants were again pressed for time. The greater attrition among women with children is also noteworthy. We speculate that these women were more challenging to follow because they had to manage more disruption in their lives and that of their children as the pandemic and its consequences waxed and waned. In addition, other pandemic-related factors not assessed may have influenced the results. Sixth, because participants in this study consented to be assigned to any of our 3 possible study conditions, our results might not generalize to women interested only in a fully self-directed program or only in a program involving a coach [49,50]. Finally, exploratory findings need to be interpreted cautiously. For these analyses, samples were smaller; the results were less consistent; and, in certain instances, the effects were smaller with wider 95% CIs than in primary analyses.

### Implications

The results of this study provide empirical support for adding the self-directed web-based SSMW treatment to the limited options available for women experiencing distress from a partner’s drinking, providing greater access to care for this large, underserved population. Although our findings suggest minimal phone coaching may facilitate engagement and incrementally improve outcomes, coach-facilitated web-based programs have additional requirements (eg, coach availability, scheduling, and training) and costs that could discourage broad implementation and increase costs to participants. As noted by Matthay et al [51] and embodied in the RE-AIM (Reach, Effectiveness, Adoption, Implementation, and Maintenance) model [52], broadly implemented treatment programs with a smaller effect size can nonetheless have a large population health impact.

Implications for future research are informed by the National Institutes of Health Stage Model [36,53]. First, future SSMW efficacy research could focus on replicating our current findings and possibly exploring features of adjunctive coaching (eg, who benefits most and optimal coaching levels). Second, while our hybrid design incorporated implementation elements, future research could focus on “...generalizability, implementation, cost-effectiveness, and social validity (acceptability to end users, program adopters, health care providers, policy makers)” [36]. Future analyses should also examine mediators of the SSMW treatment effect and secondary outcomes (eg, partner drinking, violence, and relationship stability).

Finally, the SSMW program’s cognitive behavioral approach and engaging video-rich strategies provide a framework for adapting and delivering web care for other often hidden and underserved spouse and caregiver populations. For example, eHealth adaptations of face-to-face coping skill training programs for parents of an adolescent with an SUD [54], spouses of those with a gambling disorder [55], and caregivers of a family member with an SUD [56] may accelerate research on and rapid implementation of accessible treatments for these groups.

## Appendix

Multimedia Appendix 1 CONSORT-eHEALTH checklist.

Multimedia Appendix 2 Extracted video screenshots with their related text instructions (excerpted from Stop Spinning My Wheels and formatted for this paper; portrayals are by paid actors).

Multimedia Appendix 3 Screenshot of usual web care introductory page.

Multimedia Appendix 4 Tabled descriptive statistics for primary negative affect outcomes by assessment time and study condition.

## Abbreviations

AUD: alcohol use disorder
BDI-II: Beck Depression Inventory–II
CEQ: Credibility/Expectancy Questionnaire
CONSORT: Consolidated Standards of Reporting Trials
ML: maximum likelihood
OR: odds ratio
RCT: randomized controlled trial
RE-AIM: Reach, Effectiveness, Adoption, Implementation, and Maintenance
SSMW: Stop Spinning My Wheels
STAXI-2: State-Trait Anger Expression Inventory 2
SUD: substance use disorder
UWC: usual web care
WAI-SF: Working Alliance Inventory–Short Form

## Data Availability

The data sets generated during and analyzed during this study are available from the corresponding author on reasonable request.
