# Supplementary material for: Web-Based Coping Skills Training and Coach Support for Women Living With a Partner With an Alcohol Use Disorder: Randomized Controlled Trial
Source: J Med Internet Res. 2024 Aug 29;26:e56119. doi: 10.2196/56119 (PMC11393500; doi:10.2196/56119)

Multimedia Appendix 2

This is a Multimedia Appendix to a full manuscript published in the J Med Internet Res. For full copyright and citation information see http://dx.doi.org/10.2196/jmir.56119

Extracted video screenshots with their related text instructions
(excerpted from Stop Spinning My Wheels and formatted for this paper; portrayals are by paid actors).

You started this session visiting Sue. You saw how her strong feelings of sadness, loneliness, and despair prevented her from saying anything to Ed about him not wanting to do things together. Watch to see what Sue says and does now that she's started to manage her thoughts and manage her feelings.

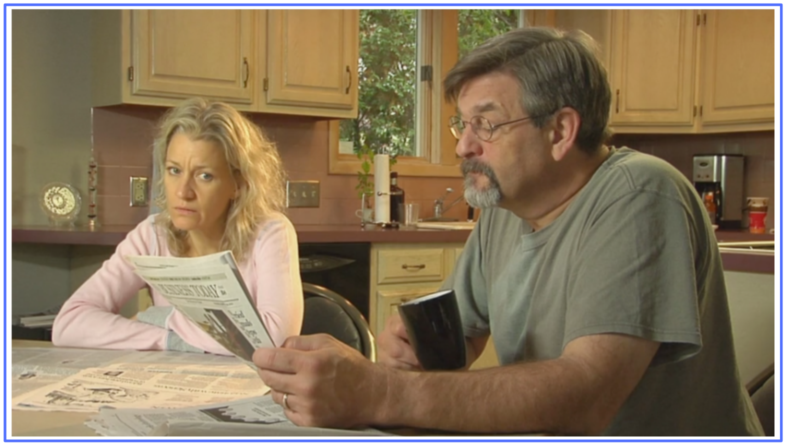

After repeated situations in which your partner says he is going to do one thing, but instead does another, particularly when it comes to his drinking, it is common for you to no longer trust what he tells you. These feelings of mistrust can linger, even if he's not drinking, and even over rather small issues. Sara finds herself in just such a situation. We'll be visiting and revisiting Sara's situation throughout this session to show how your behavior can help or hurt in getting your message across to him. Watch…

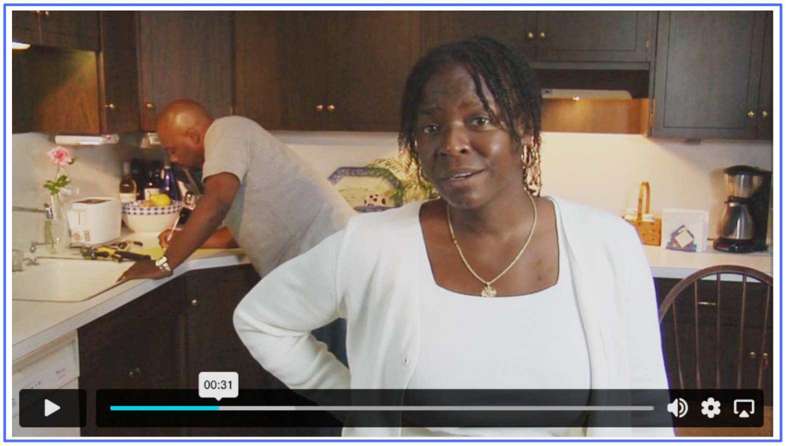

Grace is caught in a vicious cycle when it comes to Jason, and his risky behavior when drinking. To see this vicious cycle, let's look at a situation she found herself in, how she chose to deal with it, and why she kept spinning her wheels. So, watch closely…

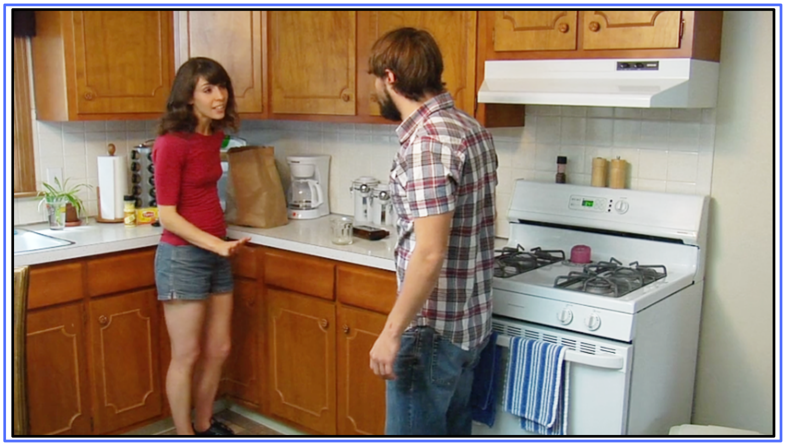

Supplement: Multimedia Appendix 2 [file jmir_v26i1e56119_app2.pdf]
