# Supplementary material for: Web-Based Coping Skills Training and Coach Support for Women Living With a Partner With an Alcohol Use Disorder: Randomized Controlled Trial
Source: J Med Internet Res. 2024 Aug 29;26:e56119. doi: 10.2196/56119 (PMC11393500; doi:10.2196/56119)
Supplement: Multimedia Appendix 3 [file jmir_v26i1e56119_app3.pdf]

Multimedia Appendix 3

This is a Multimedia Appendix to a full manuscript published in the J Med Internet Res.
For full copyright and citation information see http://dx.doi.org/10.2196/jmir.56119

Screenshot of usual web care introductory page.

Welcome, SSMW Logout

STOP SPINNING my wheels!

Sessions

1. Welcome
About the program
2. About alcohol problems
3. His drinking and you
4. What can you do?

Welcome

Welcome to the Stop Spinning My Wheels program! You've made a very important decision to change your life by joining this site.

So, Congratulations! And again, Welcome!

You know, life often is compared to a journey or road trip. It can be full of challenges, and you can't be prepared for all of them. You can run into rough, muddy roads, detours, breakdowns, and dark, stormy weather. Sometimes, you get stuck for a period of time, and just spin your wheels.

But, mixed in with all the challenges, and depending on what routes you take to deal with them, road trips can also be highly rewarding. You see and learn new things, figure out how to deal with challenges, and find beautiful, joyful places that you might not have come across otherwise.

Living with a partner who has a drinking problem, or any serious illness for that matter, can be a particularly difficult challenge on your road trip. And, you're never really prepared for it. You're taught a lot of things in school and elsewhere, but you're never taught what to expect and how to deal with a partner's drinking problem. And, often, the usual ways people cope with problems don't work when it comes to dealing with a partner who drinks too much. And after repeated tries at getting him to change don't work, you can end up feeling exhausted, and stressed out. You can feel like your life is going nowhere—you're spinning your wheels. You've spent so much energy trying to deal with his drinking problem or its consequences that you don't have the energy to put into other parts of your life.

Program goals. The main goal of this program is to provide you with information to help reduce the stress you're experiencing as a result of your partner's drinking problem. This information can help you to stop spinning your wheels, get traction, and start you on a road that's better, and healthier for you.

In the process, the information you learn may help you encourage him to change his life, too. But, face it. Although your life roads are intertwined, you don't have total control over his life, or what he does about his drinking. In the end, whether he stops drinking, cuts back, seeks out help, stays the same, or even drinks more, it is something only he can do. Still, by making changes in your life, you may encourage him to change his life, too. It's happened...

We've done some of the work for you. Making changes in your life to reduce your stress can be a challenge, and requires, as a first step, finding useful information that will help you start changing. Fortunately, a wide range of helpful information on alcohol problems, and how to cope with a partner who has a problem is out there on the Internet. But, it is not readily or easily available in one spot, and requires a lot of work finding it. So, to make it easier, this program has done the Internet search work for you, and has compiled the latest information from the Internet, and organized it for your use.

We caution that there is a large amount of information on the Internet. As with everything on the Internet, some of the information may be more accurate than others, and you will need to make the decision whether or not certain information applies personally to you, and your life—just as you would if you were doing the search on your own. When we found widely different views on the Internet about a topic, we provide you with both views. Also, while we tried to eliminate duplication, information among different sites on the Internet can be quite similar, but discussed in slightly different ways. So, you will find some repetition, because we did not want to make large changes to the information found. In fact, much of the information provided is taken, almost exactly, from the sites searched. Finally, while quite extensive, the information provided here is not absolutely everything you might find on the Internet related to alcohol problems. Should you wish to find out more information about certain topics, we provide links at the end of some sections to helpful sites.

Getting around. It's easy to get around this site. Use the directory in the left-hand column of a page to go to a topic of your choice. You don't have to go in the order that the topics appear in the directory. When you get to the end of a section, use the arrow icon to move to the next, or use the directory to go to a topic elsewhere in the site. On the top left there are two icons for you to use. The first one is a settings wheel for your profile, where you can change your phone number and email, if you need to. You can also find your password for completing research questionnaires. And the second one is a Logout arrow. To protect your privacy, we recommend that you logout and close your browser every time you leave the program. Keep coming back 24/7 to revisit topics on which you feel you need a refresher.

So, we've done the groundwork, it's now up to you. Use the information you learn on this site to start making changes in your life to reduce your stress, and stop spinning your wheels! Happy traveling!

GO TO NEXT SESSION ›

HOME CONTACT US STOP SPINNING my wheels! PRIVACY POLICY ABOUT US

© Copyright 2019, The Research Foundation for State University of New York. All Rights Reserved.
