# Supplementary material for: Web-Based Coping Skills Training and Coach Support for Women Living With a Partner With an Alcohol Use Disorder: Randomized Controlled Trial
Source: J Med Internet Res. 2024 Aug 29;26:e56119. doi: 10.2196/56119 (PMC11393500; doi:10.2196/56119)
Supplement: Multimedia Appendix 4 [file jmir_v26i1e56119_app4.pdf]

Multimedia Appendix 4

This is a Multimedia Appendix to a full manuscript published in the J Med Internet Res. For full copyright and citation information see http://dx.doi.org/10.2196/jmir.56119

Tabled descriptive statistics for primary negative affect outcomes by assessment time and study condition.

		Baseline			Posttest			6-Month Follow-Up			12-Month Follow-Up		
Outcome Measure		SSMW only	SSMW+ coach	UWC	SSMW only	SSMW+ coach	UWC	SSMW only	SSMW+ coach	UWC	SSMW only	SSMW+ coach	UWC
Depression (BDI)	M	17.0	17.1	16.2	12.3	10.0	12.8	10.7	10.7	11.4	10.7	9.6	10.7
	(SD)	(9.7)	(9.7)	(10.6)	(9.8)	(8.1)	(10.3)	(9.7)	(8.7)	(10.2)	(9.0)	(8.3)	(9.8)
	N	141	151	164	104	122	129	91	109	124	97	110	128
State Anger (STAXI)	M	21.9	20.9	20.3	18.8	18.8	19.5	19.1	18.1	19.1	19.8	17.5	18.8
	(SD)	(8.7)	(7.3)	(7.3)	(5.8)	(6.4)	(6.9)	(6.1)	(4.9)	(7.2)	(7.8)	(4.9)	(7.0)
	N	141	151	164	104	122	129	91	109	123	97	110	128

Note: SSMW: Stop Spinning My Wheels. UWC: usual web care. The sample sizes represent participants with a particular measure at each assessment period. The complete sample included 141 participants in the SSMW only condition, 151 participants in the SSMW+coach condition, and 164 participants in the UWC condition. BDI: Beck Depression Inventory-II; BDI-II diagnostic categories for depression: 0-13= minimal,14-19= mild, 20-28= moderate, 29-63= severe.; STAXI: State-Trait Anger Expression Inventory-2 State Anger (S-Ang) subscale. Statistical analysis used standard (untransformed) STAXI-2 scores.
